# Humanization of Surgical Care in the Robotic Age: A Triangular Interaction Model Between the Surgeon, the Patient, and the Technology

**DOI:** 10.3390/healthcare14142216

**Published:** 2026-07-21

**Authors:** Giuseppe Zimmitti, Maria Rosaria Portinaio, Alessandro Morandi, Paolo Terzi, Angelo Meloni, Luca Lavazza, Maria Clotilde Carra, Cinzia Ravaioli, Nicola de’Angelis

**Affiliations:** 1 Unit of General, Oncologic, Minimally Invasive and Robotic Surgery, Department of Surgery, Fondazione Poliambulanza Istituto Ospedaliero, 25124 Brescia, Italy; giuseppe.zimmitti@poliambulanza.it (G.Z.); mariarosaria.portinaio@poliambulanza.it (M.R.P.); alessandro.morandi@unimi.it (A.M.); 2 Facoltà Scienze Politiche e Sociali, Università Cattolica del Sacro Cuore, 20123 Milano, Italy; paolo.terzi@unicatt.it; 3 Direzione Sanitaria, Fondazione Poliambulanza Istituto Ospedaliero, 25124 Brescia, Italy; angelo.meloni@poliambulanza.it; 4 ASL Toscana Nord Ovest, 56124 Pisa, Italy; luca.lavazza@uslnordovest.toscana.it; 5 Department of Translational Medicine, University of Ferrara, 44121 Ferrara, Italy; mariaclotilde.carra@unife.it; 6 Azienda Ospedaliero-Universitaria di Ferrara, 44124 Ferrara, Italy; cinzia.ravaioli@ospfe.it

**Keywords:** robotic surgery, humanized surgical care, technological mediation, patient-centered care, human factors, artificial intelligence

## Abstract

**Background/Objectives**: In modern medicine, humanization of care is a central theme that highlights the complementarity of clinical care with empathy, communication, and patient-centered values. The increasing use of robotic surgery is rapidly modifying surgical practice, introducing new challenges and opportunities in preserving the human dimension of care. This review aimed to synthesize the available evidence and propose a conceptual model of humanized robotic surgical care. **Methods**: A structured narrative review of the PubMed/MEDLINE database (from inception to January 2026) was conducted using predefined keywords related to humanization of care, robotic surgery, patient perception, surgeon experience, human factors, communication, ethics, and technological mediation. Relevant English-language publications were critically synthesized to develop a conceptual framework. **Results**: The literature indicates that robotic surgery influences humanized care through three interconnected domains. First, patients frequently perceive robotic surgery as more precise and technologically advanced, which may generate unrealistic expectations and misconceptions regarding robotic autonomy. Second, robotic platforms reshape the surgeon’s experience by improving ergonomics while simultaneously modifying cognitive workload, sensory feedback, and professional identity. Third, technology itself acts as an active mediator influencing communication, trust, decision-making, and relational dynamics. Building upon these findings, we propose an original triangular conceptual framework integrating the patient, the surgeon, and the technology as three interdependent determinants of humanized robotic surgical care. The framework also provides a conceptual basis for understanding the future integration of artificial intelligence into surgical practice. **Conclusions**: Humanization of care in the era of robotic surgery requires an integrated approach that recognizes the interdependence of patient perception, surgeon experience, and technological mediation. Ensuring effective communication, supporting surgeon well-being, and preserving ethical principles will be essential to aligning innovation with patient-centered care.

## 1. Introduction

Humanization of care is a fundamental principle of modern medicine, emphasizing the centrality of the patient as a whole person rather than a mere recipient of technical interventions [1,2]. Rooted in the biopsychosocial model and further developed through patient-centered care frameworks and narrative medicine, humanized care aims to integrate clinical effectiveness with empathy, communication, relational continuity, and respect for individual values and experiences [3,4].

Over the last decades, technological innovation has profoundly transformed healthcare delivery, improving diagnostic accuracy, surgical precision, and clinical outcomes [5]. However, increasing technological complexity has also raised concerns regarding preservation of the human dimension of care, as technology progressively mediates the interaction between clinicians and patients [6].

Surgery represents a particularly complex field in this context because it is inherently invasive and frequently associated with vulnerability, fear, and dependence. Consequently, preserving communication, trust, empathy, and relational continuity throughout the perioperative pathway remains essential for maintaining humanized surgical care [7].

The introduction of minimally invasive surgery marked the first major shift toward technologically mediated interaction, reducing direct tactile contact while preserving the surgeon’s physical presence at the patient’s bedside. Robotic surgery has further transformed the surgical act through a console-based interface that introduces an additional technological layer between surgeon and patient. From the patient’s perspective, the most significant transformation is often not the surgeon’s physical location, but the perception that the robot itself performs the operation [8,9]. Such misconceptions may influence expectations, trust, and informed consent, progressively shifting perceived agency from surgeon to technology [10,11]. At the same time, robotic systems reshape the surgeon’s experience by modifying ergonomics, cognitive workload, sensory perception, and professional identity [12,13,14,15].

Within this evolving landscape, technology should not be viewed merely as a technical instrument, but as an active mediating component capable of influencing both patient perception and surgeon experience [9,10,11]. Humanization of care can therefore no longer be interpreted as a purely dyadic surgeon–patient relationship, but rather as a technologically mediated interaction involving patient, surgeon, and technology.

The aim of this narrative review is twofold. First, we synthesize the current literature addressing the impact of robotic surgery on patient perception, surgeon experience, and the humanization of surgical care. Second, we propose a conceptual triangular framework that integrates three interdependent dimensions—patient, surgeon, and technology—to better understand how humanized surgical care is shaped within increasingly technology-mediated environments. Although concepts such as patient-centered care, socio-technical systems, human factors engineering, implementation science, and artificial intelligence have substantially contributed to understanding specific aspects of healthcare delivery, they primarily address these dimensions independently (Table 1). To our knowledge, no existing conceptual model explicitly integrates the reciprocal interactions among patient, surgeon, and technology as the central determinants of humanized care in robotic surgery.

## 2. Materials and Methods

The review was designed to provide a conceptual rather than exhaustive synthesis of the literature and therefore, it is presented as a narrative review. However, it was conducted using a structured literature search of the PubMed (MEDLINE) database, performed in January 2026. Search terms combined controlled vocabulary and free-text keywords related to humanization of care, robotic surgery, and technology-mediated healthcare. The following keywords and their combinations were used: “*humanization of care*”, “*humanized care*”, “*patient-centered care*”, “*robotic surgery*”, “*robot-assisted surgery*”, “*laparoscopic surgery*”, “*surgeon experience*”, “*patient perception*”, “*human factors*”, “*human factors engineering*”, “*technological mediation*”, “*artificial intelligence*”, “*shared decision-making*”, “*surgical communication*”, “*ethics*”, “*surgeon-patient relationship*”, and “*healthcare technology*”. Boolean operators (“AND” and “OR”) were used to optimize retrieval.

The review considered original studies, systematic reviews, narrative reviews, consensus statements, clinical guidelines, and ethical or theoretical articles addressing one or more of the following topics: humanization of care; patient-centered care; robotic surgery; surgeon experience; patient perception; human factors; technological mediation; ethics of robotic surgery; and/or artificial intelligence in surgery.

Preference was given to publications written in English and to recent high-quality evidence, including systematic reviews and landmark articles. Conference abstracts, editorials without conceptual relevance, non-English publications, and articles not directly related to the objectives of the review were excluded. The retrieved literature was screened for relevance by the authors based on titles and abstracts, followed by full-text evaluation when appropriate. Because the objective of the review was to develop a conceptual framework rather than to quantitatively summarize the evidence, no formal systematic review methodology, risk-of-bias assessment, or meta-analysis was performed. Instead, the selected literature was critically interpreted and synthesized to identify the principal themes concerning patient perception, surgeon experience, technological mediation, and the humanization of robotic surgical care. These themes subsequently informed the development of the proposed triangular conceptual framework.

## 3. Humanization in Medical Care

The concept of humanization of care emerged progressively as a response to the increasing technological and organizational complexity of modern medicine. Traditionally, medical care was predominantly centered on disease-oriented and biomedical approaches, with limited attention to the patient’s subjective experience. The development of the biopsychosocial model represented a major conceptual shift by recognizing that health and illness are influenced not only by biological factors, but also by psychological, social, relational, and contextual dimensions [1].

Within this framework, humanized care extends beyond technical effectiveness alone and encompasses communication, empathy, relational continuity, patient involvement, and respect for individual values and preferences. Closely related concepts such as patient-centered care and narrative medicine have further reinforced the importance of understanding the patient not merely as a clinical case, but as a person experiencing illness within a broader personal and social context [2,3,4].

Importantly, humanization should not be interpreted as opposition to technological innovation. Rather, it concerns the ability to integrate technological progress within a care model that preserves the relational and experiential dimensions of medicine. In this sense, technology may both support and challenge humanized care depending on how it influences communication, perception, decision-making, and the therapeutic relationship [5,6,7]. This issue becomes particularly relevant in surgery, where the relationship between technical performance and human interaction is especially complex. Surgical care inherently combines high technological intensity with profound patient vulnerability, making preservation of trust, communication, and relational continuity essential throughout the perioperative process [7].

Within this evolving landscape, the progressive technological mediation introduced by minimally invasive and robotic surgery raises new questions regarding how humanization should be interpreted and maintained in increasingly interface-based surgical environments.

## 4. Evolution of Surgical Practice: From Direct Manual Interaction to Robotic Mediation

Technological innovation has progressively transformed surgical practice over the last decades, not only by modifying operative techniques but also by reshaping the way surgeons interact with patients and experience the surgical act itself. Yet this evolution should be read not as a linear technical progression, but as a complex historical process shaped by recurring tensions between innovation and the preservation of the relational dimensions of care. The transition from open surgery to minimally invasive and robotic approaches can be interpreted as a gradual evolution from direct physical interaction toward increasingly mediated forms of surgical engagement.

### 4.1. Open Surgery: Direct Gesture, Touch, and Physical Presence

Open surgery represents the traditional model of surgical interaction, characterized by direct physical engagement between surgeon and patient. In this setting, the surgeon interacts with tissues through direct manual contact, tactile feedback, and immediate visual perception of the operative field. Surgical expertise is therefore deeply connected to the embodied experience of touch, gesture, and physical proximity.

Within this environment, the surgeon’s presence remains fully integrated into the operative act. The relationship between technical performance and human interaction is immediate and continuous, as the surgeon directly experiences the patient’s anatomy, tissue resistance, and intraoperative responses. In this sense, open surgery represents the least technologically mediated form of surgical care. At the same time, open surgery also reflects the traditional symbolic image of the surgeon as a physically present and directly acting caregiver, reinforcing the perception of the surgeon as the central agent of treatment and responsibility.

### 4.2. Laparoscopy as the First Technological Discontinuity

The introduction of laparoscopic surgery marked the first major technological discontinuity in modern surgical practice [16]. By replacing large incisions with small access ports and introducing camera-guided visualization, minimally invasive surgery profoundly modified the interaction between surgeon and patient [16,17]. Compared with open surgery, laparoscopy reduced direct tactile interaction and progressively shifted the surgeon’s perception of the operative field from direct manual experience toward monitor-mediated visualization. The surgeon no longer interacted directly with tissues through the hands alone, but increasingly through instruments and visual interfaces.

Importantly, the relevance of this transformation extends beyond technical execution. The progressive introduction of technological interfaces does not merely alter surgical technique, but also modifies the experiential dimension of surgery itself. The reduction in direct sensory interaction partially transforms the embodied nature of surgical practice, while the operative act becomes progressively mediated by technology [17]. Nevertheless, despite this technological mediation, laparoscopy preserved the surgeon’s physical presence at the patient’s bedside and maintained the surgeon as the clearly identifiable central actor of care. Consequently, the traditional relational structure of surgery remained largely preserved, representing an intermediate stage between direct and interface-based surgical interaction.

### 4.3. Robotic Surgery: Interface-Based Interaction and Reconfiguration of Surgical Experience

The increasing adoption of robotic platforms represents a further stage in the technological evolution of surgery, introducing a deeper form of mediation between surgeon and patient [13,18]. In robotic procedures, the surgeon operates from a console that translates hand movements into actions performed by robotic instruments within the patient’s body. Compared with laparoscopy, robotic surgery introduces an additional technological layer that further transforms both the sensory and relational dimensions of surgical care. The surgeon’s interaction with the operative field becomes increasingly interface-based, relying predominantly on visual information because true haptic feedback remains limited. Consequently, the surgical experience progressively shifts from direct tactile engagement toward digitally mediated perception [19,20].

Importantly, the impact of robotic surgery extends beyond technical execution. Although the physical separation between surgeon and patient is generally not perceived directly by the patient intraoperatively, it substantially modifies the operative environment experienced by the surgical team and may influence the surgeon’s sense of proximity, embodiment, and engagement with the operative act. At the same time, robotic surgery may alter how patients perceive the surgical procedure itself [9,10,11]. Patients may attribute a more active role to the robotic system, potentially perceiving technology rather than the surgeon as the principal actor of care. Such misconceptions may influence trust, expectations, and informed consent [8].

This transformation therefore represents not only a technological advancement but also a progressive reconfiguration of the relationship between surgeon, patient, and technology, reshaping both the experience and the perception of surgical care.

## 5. Impact of Robotic Surgery on Patient Perception

The patient’s perception of surgical care represents a central component of humanized medicine, particularly in technologically advanced settings such as robotic surgery. Beyond clinical outcomes alone, patients interpret surgical interventions through expectations, emotions, beliefs, and perceptions that influence trust, decision-making, and overall experience of care [8,9].

### 5.1. Patient Expectations and Technological Perception

The growing diffusion of robotic surgery has progressively associated technological innovation with concepts such as precision, safety, minimal invasiveness, and superior clinical performance. Consequently, patients frequently perceive robotic procedures as inherently more advanced or effective than conventional surgical approaches, even when comparative evidence remains heterogeneous [10,11]. These perceptions are shaped not only by clinical information, but also by media representation, institutional communication, and broader social narratives surrounding technological innovation. Patient acceptance of robotic surgery is also likely to be influenced by several individual and contextual determinants. Educational level and health literacy may affect patients’ ability to understand the respective roles of the surgeon and the robotic system, whereas previous surgical experiences may shape expectations regarding the perceived benefits and risks of robotic procedures. In addition, cultural attitudes toward technology, trust in healthcare systems, and media representation of robotic surgery may contribute to substantial variability in patient perceptions across different countries and healthcare settings. These factors may partly explain the heterogeneity observed in studies investigating patient attitudes toward robotic surgery and should be considered when developing communication strategies and informed consent processes.

As a result, robotic surgery may acquire a symbolic value extending beyond its technical characteristics, becoming associated with progress, modernity, and therapeutic superiority. Within this context, patient expectations may become particularly elevated, potentially influencing satisfaction, trust, and interpretation of surgical outcomes [8,21]. Preserving alignment between patients’ expectations and clinical reality therefore represents an important component of patient-centered surgical care and requires communication strategies tailored to individual health literacy, prior experiences, and sociocultural context.

### 5.2. Misconceptions and Attribution of Agency

One of the most frequently discussed aspects of patient perception in robotic surgery concerns how patients interpret the role of the robotic system during the operative procedure. Although several qualitative studies and patient surveys have suggested that some individuals may partially attribute agency to the robotic platform, the available evidence remains relatively limited and derives primarily from observational investigations conducted in selected surgical specialties [8,9,10,21]. Importantly, this perception does not necessarily derive from misunderstanding of technical details alone, but reflects the symbolic visibility acquired by technology within robotic surgery. The robot may progressively become perceived as the central element of the procedure, while the surgeon’s role risks becoming less immediately visible within the patient’s mental representation of the operation. Patients’ perceptions of robotic autonomy should not be regarded as universal but rather as context-dependent phenomena that warrant further investigation. Future multicenter qualitative and quantitative studies will be necessary to determine the consistency of these perceptions across different clinical settings and surgical specialties.

Finally, shift in perceived agency may influence trust dynamics, expectations, and informed consent. Patients may develop unrealistic expectations regarding precision, safety, or automation, potentially attributing outcomes more to technology than to surgical expertise and clinical decision-making [10,11,12,22]. These findings highlight that robotic surgery not only transforms operative technique, but also modifies how surgical responsibility and therapeutic action are perceived within the surgeon–patient relationship.

### 5.3. Communication and Informed Consent in Technologically Mediated Surgery

In this evolving context, communication becomes increasingly important for preserving the human dimension of surgical care. Effective informed consent should not be limited to technical explanation of the procedure, but should also address patient perceptions, expectations, and misconceptions regarding robotic technology [8,12,21]. Patients should clearly understand that robotic systems function as surgeon-controlled instruments rather than autonomous operative agents. Clarifying the respective roles of surgeon and technology is essential for maintaining trust, supporting shared decision-making, and ensuring realistic expectations regarding outcomes.

More broadly, communication in robotic surgery extends beyond information transfer alone and becomes part of the relational process through which patients interpret and emotionally experience technologically mediated care. Preserving transparency, relational continuity, and patient engagement therefore remains fundamental to maintaining humanized care within increasingly technological surgical environments.

## 6. Impact of Robotic Surgery on Surgeon Activity and Perception

Robotic surgery substantially reshapes the surgeon’s experience of surgical practice by introducing both important advantages and new cognitive, technical, and organizational challenges. Beyond technical execution, robotic systems influence ergonomics, sensory interaction, cognitive processes, professional identity, and the relational dimensions of surgical care. Understanding both the benefits and the limitations of these technologies is essential for preserving humanized surgical practice.

### 6.1. Surgeon Experience: Ergonomics, Cognition, and Embodiment

Among the most consistently reported benefits of robotic surgery are improved ergonomics and reduced physical strain. Compared with open and laparoscopic procedures, robotic platforms allow surgeons to operate in a more neutral seated position with improved instrument articulation, wrist mobility, and three-dimensional visualization. Several observational studies and systematic reviews have demonstrated reductions in musculoskeletal discomfort affecting the neck, shoulders, back, and upper limbs, which represent common occupational problems among surgeons performing conventional minimally invasive surgery [23,24,25]. These ergonomic improvements may contribute not only to greater intraoperative comfort but also to improved concentration during prolonged procedures and potentially reduced cumulative physical burden associated with long surgical careers. Moreover, these ergonomic benefits are accompanied by important cognitive and perceptual changes. In robotic surgery, the surgeon interacts with the operative field through a console-based interface that translates hand movements into digitally mediated instrument actions. Consequently, the operative experience progressively shifts from direct physical interaction toward interface-based control.

Indeed, available evidence suggests that this technological evolution also modifies aspects of the surgeon’s sensory and cognitive experience. From a conceptual perspective, this transition may influence how surgeons embody the operative act and experience their interaction with patients. Traditional surgical practice is deeply connected to direct tactile feedback, physical proximity, and manual interaction with tissues. In robotic surgery, sensory perception becomes increasingly dependent on visual information, while haptic feedback remains limited [14,15,26]. As a result, surgeons must adapt to new forms of spatial perception, motor coordination, and cognitive integration. At the same time, robotic surgery may increase cognitive workload, particularly during the learning phase and in highly complex procedures. Recent human factors studies and systematic reviews have demonstrated that robotic surgery modifies rather than uniformly reduces surgeon workload, simultaneously improving ergonomics while increasing visual processing, interface interaction, and cognitive demands [11,25,27,28]. These findings suggest that robotic surgery does not simply improve surgical performance, but rather reconfigures how the surgical act is physically and cognitively experienced by the surgeon.

### 6.2. Surgeon–Patient Relationship in a Technologically Mediated Environment

Although evidence remains limited, observational studies suggest that the physical separation introduced by robotic surgery may alter team communication and the surgeon’s perceived proximity to the patient, thereby reinforcing the importance of preoperative and postoperative interactions [28,29].

The introduction of robotic systems modified the environment in which the surgeon–patient relationship develops. In robotic procedures, the surgeon operates from a console physically separated from the patient during the operation. Although this separation is not perceived directly by the patient intraoperatively, it substantially changes the operative environment experienced by the surgical team [28] and may influence the surgeon’s sense of physical proximity and engagement with the operative act. Importantly, the surgeon–patient relationship extends well beyond the intraoperative setting itself. Trust, communication, and shared decision-making are primarily established during preoperative and postoperative interactions, which therefore acquire even greater importance within technologically mediated surgery [12].

In this context, communication becomes essential not only for explaining technical aspects of robotic procedures, but also for preserving relational continuity and reinforcing the surgeon’s role as the primary agent of care. Maintaining visibility of the surgeon’s expertise and responsibility is particularly important in environments where technology risks becoming symbolically dominant within the patient’s perception of the procedure [29]. These aspects highlight that the human dimension of surgery depends not only on physical proximity, but also on preservation of communication, trust, and relational engagement throughout the perioperative pathway.

### 6.3. Professional Identity, Responsibility, and Surgeon Well-Being

Emerging qualitative studies further suggest that interface-based surgery may progressively reshape surgeons’ professional identity by shifting emphasis from manual dexterity towards technological proficiency, cognitive integration, and systems management [29,30,31].

The progressive adoption of robotic surgery also has important implications for surgical education and mentorship. Traditional surgical training has historically relied on direct observation, graduated operative autonomy, tactile feedback, and close physical interaction between trainee and mentor. Robotic surgery partially modifies these educational dynamics by introducing console-based operating, digital interfaces, and simulation-based learning. Although robotic platforms provide valuable opportunities for structured skill acquisition, objective performance assessment, and procedural simulation, they also require adaptation of mentorship models to ensure that trainees develop not only technical competence but also communication skills, professional judgment, teamwork, and ethical responsibility.

Despite increasing technological mediation, responsibility for surgical decision-making and patient safety remains firmly centered on the surgeon. Maintaining clarity regarding accountability and professional responsibility therefore remains essential within robotic surgery, particularly in contexts where patients may overestimate the autonomy of technological systems [30,31]. In addition, attention to surgeon well-being is increasingly important within technologically intensive surgical environments. Although robotic systems may reduce physical strain, the cognitive demands associated with prolonged interface-based interaction, technological complexity, and high-performance expectations may contribute to mental fatigue and burnout [25,27,28]. These considerations highlight that preservation of humanized surgical care should also include attention to the surgeon’s physical, cognitive, and emotional experience, recognizing clinician well-being as an important component of quality of care.

## 7. The Triangle of Humanization in Robotic Surgery: A Conceptual Framework

The progressive integration of robotic systems into surgical practice has transformed not only technical execution but also the relational dynamics through which surgical care is experienced and delivered. The findings discussed throughout this review suggest that humanization in robotic surgery can no longer be interpreted exclusively through the traditional dyadic relationship between surgeon and patient. Instead, robotic surgery introduces a third active component represented by technology itself, which increasingly mediates interaction, perception, communication, and operative experience.

**Figure 1:**
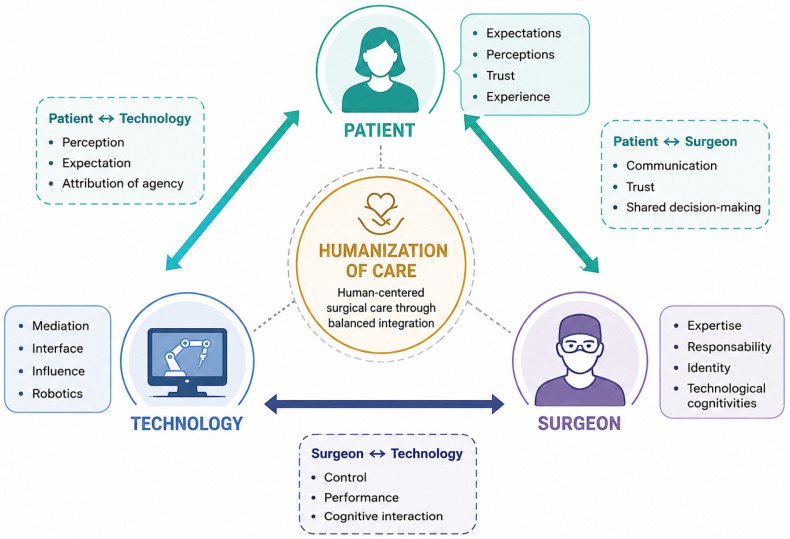
Schematic representation of the conceptual framework in which humanized surgical care is integrated as the dynamic interaction between the patient, the surgeon, and the technology.

Within this evolving context, we propose a conceptual framework in which humanized surgical care is understood as the dynamic interaction between three interconnected dimensions: the patient, the surgeon, and technology (Figure 1). The patient dimension includes expectations, emotional responses, trust, perception of the procedure, and interpretation of the surgeon’s role. As discussed, robotic surgery may influence how patients attribute agency, understand responsibility, and emotionally experience technologically mediated care [8,9,10,11,12].

The surgeon dimension encompasses not only technical expertise, but also cognitive workload, ergonomics, embodiment, professional identity, and relational engagement. Within the conceptual framework proposed here, technology is conceptualized as an active mediating interface capable of influencing perception, communication, and relational dynamics between surgeon and patient [20,21,23,24,25,26,27,28].

Importantly, the quality of humanized surgical care depends not only on the individual characteristics of each component, but also on the balance of their interactions. The patient–surgeon relationship remains central for trust and communication; the surgeon–technology interaction influences operative performance and cognitive experience; and the patient–technology relationship shapes expectations, symbolic perception, and interpretation of the surgical process. Within this framework, technological innovation should not be viewed as inherently opposing humanization. Rather, the challenge lies in integrating increasingly complex technologies while preserving communication, trust, accountability, and relational continuity throughout the perioperative pathway.

Unlike existing patient-centered or technology-centered approaches, the proposed triangular framework explicitly conceptualizes humanized surgical care as the dynamic balance between three interacting actors, thereby providing an integrative perspective through which technological innovation and human relationships can be considered simultaneously rather than independently.

The proposed framework may also facilitate interpretation of everyday clinical situations encountered during robotic surgical practice. For example, during the preoperative consultation, a patient may believe that the robot performs the operation autonomously. Within the triangular framework, this situation reflects an imbalance in the patient–technology relationship that should be corrected through communication reinforcing the surgeon’s central role. Similarly, during the learning phase of robotic surgery, increased cognitive workload experienced by the surgeon may temporarily influence communication with the surgical team or the patient. In this case, the framework highlights the interaction between surgeon and technology and emphasizes the importance of training, mentoring, and human factors to maintain high-quality care. Finally, future AI-assisted decision-support systems may recommend specific operative strategies. The framework emphasizes that such recommendations should support rather than replace clinical judgment, thereby preserving surgeon accountability while maintaining patient trust and transparency (Table 1).

### Emerging Artificial Intelligence and Future Challenge of Humanized Surgical Care

Robotic surgery is currently undergoing a second technological transformation driven by the rapid integration of artificial intelligence (AI), machine learning, computer vision, and advanced decision-support systems. Although contemporary robotic platforms remain entirely surgeon-controlled, AI-assisted technologies are increasingly being developed to support surgical planning, anatomical recognition, workflow optimization, instrument guidance, performance assessment, and intraoperative decision-making [32,33]. These innovations have the potential to improve precision, efficiency, and surgical safety while reducing cognitive workload during complex procedures.

At present, autonomous robotic surgery remains largely experimental, with most commercially available systems functioning as telemanipulation platforms under the direct control of the surgeon. Nevertheless, increasing levels of automation may progressively modify how responsibility, trust, and clinical decision-making are distributed between surgeons and intelligent systems. Questions regarding algorithm transparency, explainability, accountability, data governance, and medico-legal responsibility are therefore becoming increasingly relevant.

Within this evolving landscape, the proposed triangular framework remains applicable but also acquires greater significance. As artificial intelligence becomes progressively integrated into robotic surgery, technology should no longer be considered merely a passive interface but rather an increasingly adaptive and interactive component capable of influencing clinical decisions, workflow organization, and surgeon–patient communication. Preserving the human dimension of surgical care will therefore require maintaining the surgeon as the principal decision-maker while ensuring that AI functions as an assistive technology that augments, rather than replaces, human expertise, empathy, and ethical responsibility.

A further challenge associated with the increasing adoption of robotic surgery concerns equitable access to technological innovation. Although robotic platforms have expanded considerably over the past two decades, their availability remains highly heterogeneous across countries, healthcare systems, hospitals, and socioeconomic settings. Access to robotic surgery may therefore depend not only on clinical indications but also on institutional resources, geographical location, healthcare infrastructure, reimbursement policies, and patients’ socioeconomic status. Such disparities raise important ethical questions because unequal access to advanced surgical technologies may inadvertently contribute to widening existing healthcare inequalities.

Cost-effectiveness also represents an important consideration. Although robotic surgery may improve ergonomics, facilitate technically demanding procedures, and potentially reduce selected postoperative complications, its substantial acquisition, maintenance, and training costs continue to limit widespread implementation. Consequently, decisions regarding adoption of robotic platforms should consider not only technical performance but also long-term clinical value, sustainability, and equitable resource allocation. Future economic evaluations incorporating patient-reported outcomes, surgeon well-being, and organizational benefits may provide a more comprehensive assessment of the overall value of robotic surgery within different healthcare systems.

## 8. Discussion

The present review highlights how the progressive integration of robotic technologies into surgical practice has transformed not only technical performance but also the human dimensions of surgical care. While robotic platforms consistently improve visualization, dexterity, precision, and ergonomics, their influence extends beyond operative outcomes, affecting patient perceptions, surgeon experience, communication, and the relational dynamics that characterize the perioperative pathway [8,9,10,11,12,28,29]. Overall, the available evidence suggests that robotic surgery modifies human interactions in ways that cannot be fully explained by its technical advantages alone. Nevertheless, much of the current literature remains observational, qualitative, or survey-based, highlighting the need for prospective studies evaluating patient-reported experience measures, surgeon well-being, communication quality, and shared decision-making in technologically mediated surgical environments.

The principal contribution of this review lies in proposing an original conceptual framework for understanding humanized care in robotic surgery. Although patient-centered care, socio-technical systems, human factors engineering, and, more recently, human-centered artificial intelligence have each contributed substantially to healthcare research, these approaches generally address individual components of healthcare delivery. Patient-centered care primarily emphasizes communication, empathy, shared decision-making, and patient experience. Socio-technical systems focus on interactions between humans, technologies, and organizational processes. Human factors engineering seeks to optimize safety, usability, and system performance, whereas human-centered AI aims to ensure that intelligent systems remain transparent, trustworthy, and supportive of human decision-making. Rather than replacing these complementary perspectives, the triangular framework proposed here integrates them into a unified conceptual model specifically adapted to robotic surgery by explicitly considering the patient, the surgeon, and the technology as three interdependent actors whose reciprocal interactions determine the quality of humanized surgical care.

Existing models rarely consider technology as an active relational component capable of simultaneously influencing patient perceptions, surgeon cognition, communication, trust, professional identity, and therapeutic relationships. As robotic surgery progressively evolves towards increasingly intelligent and interconnected systems, understanding these reciprocal interactions becomes essential for preserving the human dimension of care.

Thus, the proposed framework contributes to the broader discussion surrounding humanized care in the era of digital medicine. Traditionally, humanization has been understood as preserving empathy, communication, trust, and relational continuity despite increasing technological complexity. However, the ongoing digital transformation of healthcare—including robotic surgery, telemedicine, artificial intelligence, digital decision-support systems, and remote healthcare—has progressively altered the context in which these relationships develop. Humanized care should therefore no longer be interpreted solely as the quality of the patient–clinician relationship but also as the ability to preserve human values while interacting with increasingly sophisticated technologies. Within this perspective, technology should not be viewed as inherently dehumanizing; rather, its influence depends on how it is designed, implemented, and integrated into clinical practice.

This broader perspective is also consistent with current theories of technology acceptance. Existing technology acceptance models have traditionally focused on healthcare professionals, emphasizing perceived usefulness, usability, trust, and willingness to adopt innovative technologies. Robotic surgery illustrates that technology acceptance extends beyond clinicians to include patients, whose expectations, beliefs, and perceptions of robotic systems may substantially influence informed consent, shared decision-making, trust, and satisfaction. The available evidence suggests that some patients may attribute a greater operative role to robotic systems than is technically justified; however, this phenomenon remains supported primarily by qualitative studies and surveys, and its prevalence across different surgical specialties and healthcare settings remains uncertain. Consequently, patient attribution of agency should be regarded as an emerging and context-dependent phenomenon that warrants further empirical investigation rather than an established characteristic of robotic surgery.

Similarly, the proposed framework complements traditional human factors engineering by extending its scope beyond technical performance and patient safety to include relational and ethical dimensions of care. Human factors approaches primarily aim to optimize interactions between humans and technology in order to improve efficiency, usability, and safety. Our framework suggests that successful integration of robotic surgery also requires preserving communication, professional identity, accountability, empathy, and patient trust. In this sense, technology should be considered not only as an operative tool but also as an active mediator of the therapeutic relationship.

The review also highlights the importance of considering both the benefits and challenges experienced by surgeons. Current evidence consistently demonstrates that robotic surgery improves ergonomics, reduces musculoskeletal strain, and facilitates technically demanding procedures through enhanced visualization, tremor filtration, and instrument articulation [23,24,25,26,27,28]. These advantages may contribute to improved occupational health, greater procedural comfort, and potentially longer professional careers. Conversely, robotic surgery also redistributes workload by shifting demands from predominantly physical tasks towards increased cognitive processing, interface interaction, and technological management. Rather than representing a simple increase or decrease in workload, robotic surgery appears to modify its nature, requiring surgeons to develop new cognitive, perceptual, and communicative competencies alongside traditional technical expertise. Maintaining surgeon well-being should therefore be regarded as an essential component of humanized care, recognizing that clinician health, professional satisfaction, and occupational sustainability ultimately contribute to patient safety and quality of care.

The rapid integration of artificial intelligence further reinforces the relevance of the proposed framework. Although currently available robotic systems remain entirely surgeon-controlled, emerging technologies incorporating machine learning, computer vision, predictive analytics, and decision-support algorithms are progressively changing surgical practice [33,34]. These innovations may improve precision and efficiency while simultaneously introducing new ethical questions concerning transparency, explainability, accountability, autonomy, data governance, and equitable access to advanced technologies. Consequently, preserving humanized care will increasingly depend on maintaining the surgeon as the principal clinical decision-maker with human expertise, empathy, and professional responsibility.

The emergence of adaptive human–robot collaboration, digital twins, mixed reality, and increasingly intelligent robotic platforms further supports the relevance of the proposed framework [35]. Rather than viewing robotic surgery as a static technology, these developments suggest that future surgical environments will involve continuous interaction between surgeons and increasingly adaptive systems. Consequently, preserving humanized care will depend not only on technical innovation but also on maintaining transparency, human oversight, trust, and effective communication as human–robot collaboration becomes progressively more sophisticated.

From a clinical perspective, the proposed framework has several practical implications. Communication strategies should explicitly address common misconceptions regarding robotic surgery and clarify the surgeon’s central role throughout the operative process. Surgical education should evolve by incorporating communication skills, cognitive ergonomics, human factors, and ethical reasoning alongside technical training. Furthermore, healthcare organizations and technology developers should ensure that robotic systems are implemented in ways that support rather than undermine clinician–patient relationships. The framework may also provide a useful conceptual basis for evaluating future innovations in digital surgery and AI-assisted healthcare.

This review has several limitations. First, as a narrative review, it does not follow the methodological procedures of a systematic review and therefore remains susceptible to selection bias in the identification and interpretation of the available literature. Although the literature search was conducted using a structured strategy, no formal protocol, risk-of-bias assessment, or quantitative synthesis was performed. Consequently, the conclusions presented should be interpreted as a conceptual synthesis rather than a comprehensive evaluation of the available evidence. Second, many of the considered studies were observational, qualitative, or survey-based, reflecting the emerging nature of research on humanization in robotic surgery. As a result, several concepts discussed—including patient perception, professional identity, and technology-mediated relationships—remain difficult to quantify objectively and may vary across healthcare systems, surgical specialties, and cultural contexts. Accordingly, the proposed triangular framework should be considered a theoretical model intended to facilitate understanding and generate future hypotheses rather than a validated explanatory model. Finally, given the rapid evolution of robotic surgery and artificial intelligence, the proposed framework should be regarded as dynamic and may require refinement as new empirical evidence becomes available.

The proposed framework also complements implementation science models, which primarily focus on organizational determinants influencing implementation of healthcare innovations. Unlike these approaches, our model specifically emphasizes the relational interactions among patients, surgeons, and technology within the surgical environment.

Future multidisciplinary research should therefore focus on validating the proposed framework across different surgical specialties and healthcare systems, exploring its applicability to other technology-mediated healthcare settings, and developing practical tools capable of evaluating its impact on patient experience, clinician well-being, communication quality, technology design, and the ethical implementation of increasingly intelligent surgical systems.

## 9. Conclusions

The triangular framework proposed in this review attempts to integrate the dimensions that are individually considered in the current literature in a holistic approach, by conceptualizing humanized surgical care as the dynamic interaction between patient, surgeon, and technology. Within this model, technology should be viewed as an active mediating component whose integration must remain balanced and ethically grounded.

Humanization of care depends on preserving communication, trust, accountability, relational continuity, and clinician engagement within increasingly mediated surgical environments [2,5,6,7].

From a clinical perspective, several implications emerge:

- Communication strategies should specifically address common misconceptions regarding robotic surgery and clarify the surgeon’s central role within the operative process [7,12].
- Surgical training programs should also incorporate cognitive, perceptual, and human factors alongside technical proficiency, recognizing that robotic surgery requires adaptation not only of operative skills but also of relational and cognitive dimensions of practice [28,29,30,31].
- Attention to surgeon well-being is also increasingly important. Although robotic surgery may improve physical ergonomics, the cognitive demands associated with technologically intensive environments may contribute to mental fatigue and burnout, with potential implications for clinician performance and quality of care [25,27].

From a broader research perspective, the continuing expansion of resource-intensive technologies such as robotic surgery raise several questions about:

- Equity and access to care, particularly in healthcare systems where availability remains limited or unevenly distributed.
- Progressive integration of artificial intelligence, advanced automation [22], and telesurgical platforms [32,33] could introduce new forms of interaction and decision-making within surgical care. Although these innovations are not yet fully integrated into routine clinical practice yet, they may raise additional ethical and relational challenges concerning autonomy, accountability, data governance, and preservation of the human dimension of care [34].

Future multidisciplinary research will therefore be required to better explore how technological innovation may influence patient experience, surgeon well-being, communication, and relational dynamics within modern surgical care. Moreover, prospective validation studies using qualitative and quantitative methodologies will be required before the proposed framework can be considered applicable to routine clinical practice.

## Figures and Tables

**Table 1:** Comparison between existing conceptual frameworks and the proposed triangular model of humanized robotic surgical care.

Framework	Primary Focus	Role of Technology	Main Actors	Main Limitation in Robotic Surgery	Added Value of the Proposed Triangular Model
**Patient-centered care**	Communication, empathy, shared decision-making, patient values	Passive tool supporting care	Patient and clinician	Limited consideration of technology as an active relational component	Integrates technology as a determinant influencing both patient and surgeon
**Socio-technical systems**	Interaction between people, technology and organization	Component of the healthcare system	Humans, technology, organization	Focuses mainly on organizational performance rather than the therapeutic relationship	Specifically addresses the relational dynamics of robotic surgical care
**Human factors engineering**	Safety, usability, ergonomics, performance	Interface to optimize human performance	Clinicians and technology	Emphasizes technical performance more than patient experience	Combines technical, cognitive and relational dimensions of care
**Human-centered AI**	Trustworthy, transparent and explainable AI	Intelligent decision-support system	Human users and AI	Primarily focused on AI rather than the overall surgeon–patient relationship	Places AI within a broader framework of humanized surgical care
**Proposed triangular model**	Humanization of robotic surgical care	Active mediator of communication, perception and relationships	Patient–surgeon–technology	—	Integrates the reciprocal interactions among the three actors within a single conceptual framework specifically developed for robotic surgery

## Data Availability

No new data were created or analyzed in this study. Data sharing is not applicable to this article.
